# Diterpene Biosynthesis in Rice Blast Fungus *Magnaporthe*

**DOI:** 10.3389/ffunb.2022.869823

**Published:** 2022-04-12

**Authors:** Ayousha Shahi, Houlin Yu, Sibongile Mafu

**Affiliations:** ^1^Plant Biology Graduate Program, University of Massachusetts-Amherst, Amherst, MA, United States; ^2^Department of Biochemistry and Molecular Biology, University of Massachusetts-Amherst, Amherst, MA, United States

**Keywords:** diterpene synthase, Ascomycete, *Magnaporthe*, manoyl oxide, pimaradiene

## Abstract

Plant-pathogenic fungi harbor various specialized metabolites including diterpenoids that function as hormones and virulence factors. The fungus *Magnaporthe oryzae* is the causal agent of rice blast disease and can infect over fifty grass species. We demonstrate that rice blast fungi encode two diterpene synthases that produce normal pimara-8,15-diene and manoyl oxide scaffolds. Phylogenetic analysis of diterpene synthases among rice blast pathotypes showed functional conservation of these two core diterpene synthases amongst all pathotypes and suggests further expansion in those infecting select grass species. These insights into the blast fungal terpenome may inform efforts to counteract deleterious phytopathogens in crucial food crops.

## Introduction

Plant pathogenic fungi in the phylum *Ascomycota* produce a diverse array of specialized metabolites with versatile roles that confer a competitive advantage in nature regarding virulence, interspecies communication and competition, and biotic and abiotic stress protection (Quin et al., 2014). Diterpenoids are one such class of metabolites that have various bioactive roles in both plants and fungi (Roncal et al., 2002; Tudzynski, 2005; Bromann et al., 2012). Labdane-related diterpenoids represent more than 7,000 molecules but only a handful have been characterized in fungal organisms to date. Generation of these diterpene scaffolds is a two step-process mediated by class II and class I diterpene synthases that catalyze cyclization of the precursor substrate (*E,E,E*)-geranylgeranyl diphosphate (GGPP) (Peters, 2010). Class II diterpene synthases catalyze a protonation-initiated cyclization of GGPP, generating stereospecific copalyl diphosphate (CPP) intermediates. Class I diterpene synthases carry out ionization-dependent cyclization of CPP resulting in the further elaborated structural diversity of the diterpene scaffolds. However, most diterpene synthases in fungi have fused both class II and class I activities into a single bifunctional enzyme. Terpene synthases in Ascomycete are thought to have occurred through horizontal gene transfer of a diterpene synthase from plants to fungi. The resultant chemical diversity stems from evolutionary duplication and neofunctionalization of an ancestral diterpene synthase (Cao et al., 2010; Fischer et al., 2015).

*Pyricularia oryzae* (*syn Magnaporthe oryzae*), also known as rice blast fungus, destroys 10–30% of total rice yield annually worldwide. Other than rice, the fungus infects over fifty grass species, the most alarming being its recent discovery in wheat (Wilson and Talbot, 2009; Zhang et al., 2016; Fernandez and Orth, 2018). Rice plants, in response to infection by rice blast fungus, induce accumulation of several defense-related diterpenoids, such as momilactones A and B, and phytocassanes A – E (Peters, 2006). Interestingly, the *Magnaporthe* genome also harbors two diterpene synthases that have been demonstrated to be transcriptionally upregulated in various rice and fingermillet samples both in nutrient media and field isolates (Chiapello et al., 2015; Saha et al., 2020, 2021). However, the products of these diterpene synthases are unknown. Hypothesizing that these two diterpene synthases may have a role during plant infection or environmental adaptation, we report here biochemical characterization of these two diterpene synthases, -MoDiTPS1 & MoDiTPS2, from the rice blast fungus *Magnaporthe oryzae*. We discuss the phylogenetic evolution of diterpene biosynthesis in the fungal terpenome and provide evidence for the intermediate diterpene metabolites produced by *Magnaporthe*.

## Methods

### Diterpene Synthase Mining and Phylogenetic Analysis of *M. oryzae* Diterpene Synthases

Genome assemblies of 52 *Magnaporthe oryzae* strains were downloaded from NCBI and their gene/protein models were predicted using Augustus/3.3.2 that was trained using *Magnaporthe grisea* gene model (Chiapello et al., 2015; Gladieux et al., 2018). A BLAST library of proteomes of all strains were made, and protein sequences of seven diterpene synthases in Chiapello et al. (2015) (five from the BR32 genome and two from the 70–15 genome from NCBI- MGG_01949 and MGG_14722) were queried against the library using blastp (blast/2.7.1+) (Chiapello et al., 2015; Gladieux et al., 2018). The resulting matching sequences were filtered by retaining the matches with specific parameters (identity >30% and e value <1e-20). The filtered sequences were taken as the query again and searched against the BLAST library and the same filtering was applied. The process was iterated three times until no more matching sequences was found. This finally resulted in 321 protein sequences that were further annotated as DiTPS candidates in 52 *Magnaporthe oryzae* strains. Sequences <600 amino acids were not included in the study. The putative DiTPSs were further checked for the presence of functional catalytic sites (class I and class II active sites) to shortlist bifunctional diterpene synthases. Class II cyclases are characterized by the DxDD motif involved in protonation-initiated cyclization. Class I active site has the characteristic D(D,E)xx(D,E) motif that facilitates ionization initiated cyclization of the class II intermediate (Supplementary Figure 1). A summary of the computational pipeline is depicted (Supplementary Figure 2). For phylogenetic analysis, 14 genomes were selected representing two genomes each from seven pathotypes (Supplementary Table 1). Similarly, for sesquiterpene synthases, the blast search was iterated three times (identity >30% and e value <1e-20) until no more matching sequences were found. This finally resulted in 160 protein sequences and the sequences with motifs- DDxxD, NSE/DTE, and RxR and sequence lengths more than 220 amino acids were used for the phylogenetic analysis. However, for chimeric terpene synthases, iterated blast search with identity >30% didn't result in a limited pool, and therefore a stringent parameter of identity >90% was used and the blast search was iterated four times (e value <1e-20) until no more matching sequences were found. This finally resulted in 194 protein sequences and the sequences with N-terminal motifs- D/ED/ExxD/E, NSE [N/HDxx(S/T)xxxD/E], and C-terminal motifs- DDxxD and DDxxN sequence length more than aa were used for phylogenetic analysis.

The filtered sequences for all three terpene synthases- di-, sesqui, and chimeric terpene synthases were then aligned using Muscle alignment tool followed by construction of maximum likelihood tree with 100 bootstrap repetitions using the software MEGAX (Kumar et al., 2018).

### Cloning

Synthetic codon-optimized diterpene synthase genes for heterologous expression in *Escherichia coli* were obtained from Thermo Scientific. The genes were amplified by PCR and sub-cloned into pET28b expression vectors between Bam HI and Hind III restriction sites, using In-fusion HD cloning kit (Takara Bio) and primers in Supplementary Table 2. The constructs were confirmed by full-length sequencing. The genes were also cloned into pESC-URA vector between Not I and Spe I restriction sites using In-fusion HD cloning kit and primers for expression in yeast (Supplementary Table 2). The diterpene synthase Class I knock-out mutants were generated by mutating the first aspartate residue of the DDxxD class I motif to alanine by PCR based site-directed mutagenesis of pET28b vectors cloned with diterpene synthase genes (Supplementary Table 2).

### Enzymatic Analysis by Combinatorial Expression in *E. coli*

The diterpene synthase products were determined using a modular metabolic engineering system (Cyr et al., 2007; Morrone et al., 2010). Briefly, the vectors carrying each diterpene synthase gene were individually co-transformed with a GGPP synthase carried on a compatible pACYC-Duet plasmid into C41 Overexpress *E. coli* cells pre-transformed with pIRS (to increase yield) for recombinant expression of each diterpene synthase. The resulting recombinant strains were grown at 37°C overnight in 5 ml of TB media (supplemented with 100 mM phosphate buffer pH 7.5 and appropriate antibiotics). One ml of overnight culture was used to inoculate 50 ml of TB media and the cultures were incubated at 37°C and 160 rpm till mid-log phase. At this point, the temperature was dropped to 16°C for 1 h and then 1 mM isopropylthiogalactoside (IPTG) was added for induction and 50 mM sodium pyruvate was added as a carbon supplement. The induced culture was further incubated for 3 days at 16°C and 160 rpm to allow product formation. The resulting products were extracted adding equal volume of hexane and the organic layer was separated, dried and re-dissolved in 1 ml of n-hexane for product analysis.

### Enzymatic Analysis in Yeast

The pESC-URA vectors carrying diterpene synthase genes were co-transformed with mFPS plasmid (avian FPP mutant expressing GGPP) into yeast ZXB competent cells using previously described (Zhuang and Chappell, 2015). The yeast cells were grown and maintained in YPD media supplemented with ergosterol. The positive colonies were obtained using appropriate SC selection media containing ergosterol (SCE media) and were used to inoculate 50 ml of SCE media containing galactose as an inducer for pESC-URA cloned genes. The culture was grown at 28°C and 220 rpm for 6 days before extraction of resultant products using equal volume of acetone, followed by one volume of hexane. The upper organic layer was centrifuged, dried up and redissolved in 1 ml of n-hexane for product analysis.

### Metabolite Analysis by GC-MS

The product analysis was performed by gas chromatography with mass spectral detection (GC-MS) on Agilent 7890/7000C GC-QQQMS using an Agilent HP-5MS column with 1.2 ml/min helium flow rate. Samples (1 ul) were injected in splitless mode using an Agilent 7693 autosampler. The following temperature program was used: the oven temperature started at 70°C, which was maintained for 1 min, and then increased at a rate of 20°C/min to 300°C, where it was held for another 2 min. The mass spectrum was recorded by mass-to-charge ratio (*m/z*) values in the range from 60 to 600 starting from 9 min after sample injection until the end of the run. For identification of enzymatic products, the retention time and mass spectra of products were compared to authentic standards (Supplementary Table 3).

### *In silico* Analysis of Putative Biosynthetic Gene Clusters

The diterpene scaffolds generated by diterpene synthases are further decorated by oxidases, reductases, and transferases to form bioactive diterpenoids. The biosynthetic genes of specialized metabolites in most fungi are typically located next to each other and form biosynthetic gene clusters. To evaluate if MoDiTPS1 and MoDiTPS2 occur as functional biosynthetic gene clusters (BGCs) in *M. oryzae* genomes, putative BGCs of MoDiTPS1 and MoDiTPS2 were predicted using *in-silico* analysis tools. Briefly, the putative biosynthetic gene clusters of the two diterpene synthases -MoDiTPS1 and MoDiTPS2 from *M. oryzae 70-15* reference genome were first predicted through sorting of genes with oxidase, reductase, transferase, or GGPP synthase annotations, 70 kb upstream and downstream of diterpene synthase genes from *Mo 70-15* genome. The synteny of these putative biosynthetic genes among other pathotype genomes was then analyzed by using the sorted genes as query against chromosomes containing diterpene synthase genes of *B71*, and *MZ5-1-6* genomes using SimpleSynteny online tool (Veltri et al., 2016).

## Results

### Phylogenetic Analysis of MoDiTPSs Among *M. oryzae* Pathotypes Reveals an Expansion of Diterpene Synthases

**Figure 1:**
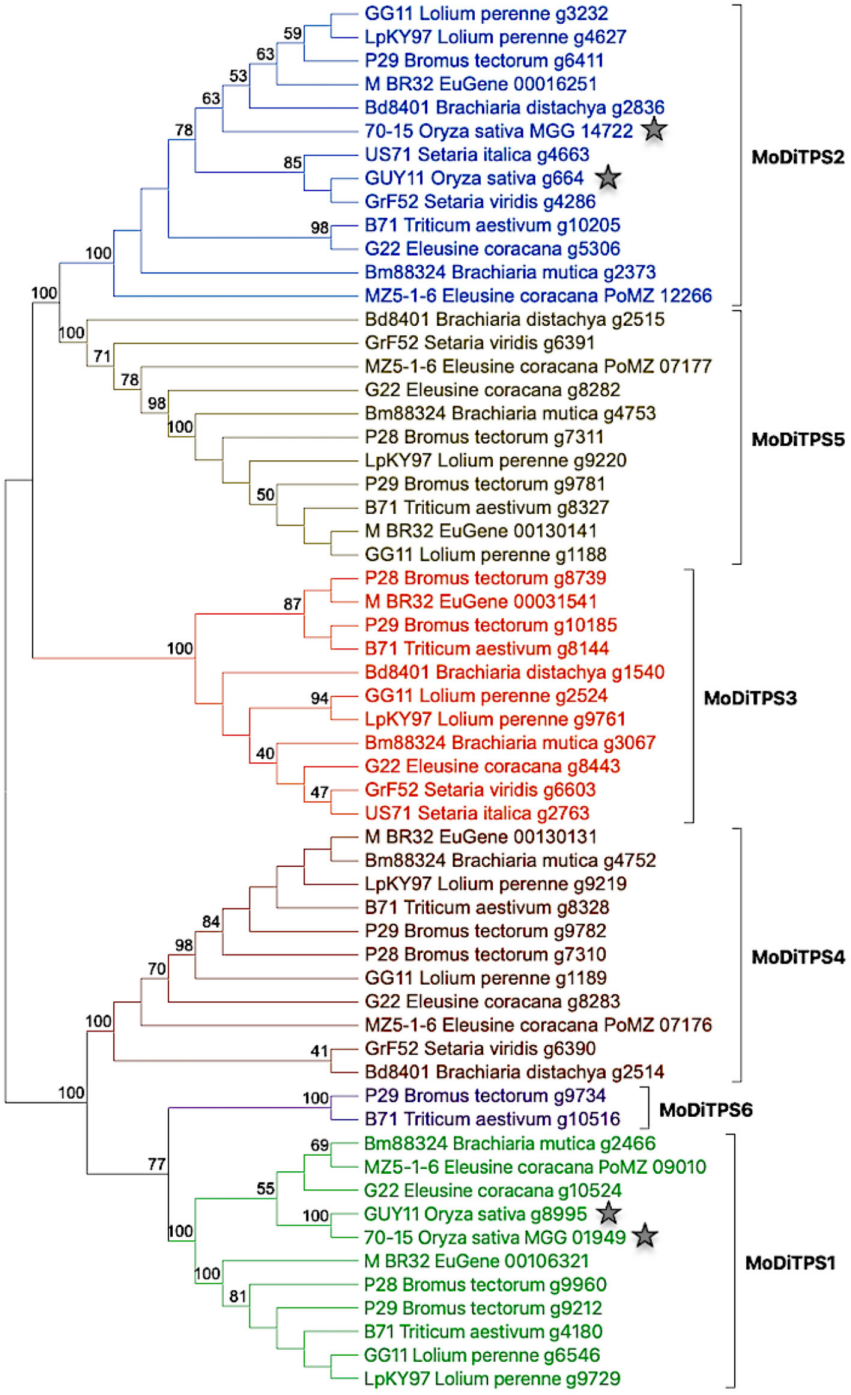
phylogeny based expansion of diterpene synthases in *Magnaporthe oryzae* pathotypes. Phylogenetic tree showing relationship among 59 DiTPS enzymes belonging to 14 *M. oryzae* pathotypes. The DiTPS are named by their genome name followed by their host plant and annotation number. The diterpene synthases of rice pathotypes are indicated with a star to illustrate relative expansion of DiTPSs in other grass pathotypes. The details of genomes and DiTPSs present in them are illustrated in Supplementary Table 1. The sequences were aligned using Muscle alignment tool and maximum likelihood tree was constructed using MEGAX software with default parameters and 100 bootstrap repetitions.

Given the potential roles of diterpenoids in fungal development and manipulation of host defense mechanisms, we assessed the genetic potential of the *M. oryzae* reference genome (Mo70_15) to produce diterpenes. Using BLAST analysis, and presence of characteristic protein domains for published genome analysis we identified two putative diterpene synthases (Chiapello et al., 2015). As *Magnaporthe* is a species complex that infects other grass species in addition to rice, we then investigated the distribution of diterpene synthases (DiTPSs) among *M. oryzae* pathotypes that infect different host plants. Utilizing the diterpene synthase mining approach described in the Methods section and depicted in Supplementary Figure 2, we identified putative DiTPSs from 52 *M. oryzae* proteomes covering 14 pathotypes (Chiapello et al., 2015; Gladieux et al., 2018). As most bifunctional DiTPS sequences are >800 amino acids, sequences <600 amino acids were not included in the study. The putative DiTPSs were further checked for the presence of functional catalytic motifs (in both class I and class II active sites) to create a shortlist of bifunctional diterpene synthases. This DiTPS mining approach revealed presence of an additional enzyme—DiTPS6 in addition to five annotated DiTPS enzymes. The DiTPSs are numbered as MoDiTPS1 through MoDiTPS6. The phylogenetic distribution of DiTPSs among 14 representative pathotypes is illustrated in Figure 1.

There is an increased number of DiTPS enzymes in pathotypes of grass species, such as wheat (*Triticum*), brome grass (*Bromus*), and fingermillet *(Eleusine*) compared to rice (*Oryza*) pathotype. MoDiTPS6 is uniquely present in the pathotypes from two grasses –*Triticum* and *Bromus*. The observed expansion of DiTPS enzyme family aligns with several studies that have highlighted the fluidity of fungal genomes and expansion of various gene families (Haas et al., 2009; Duplessis et al., 2011). Phylogenetic analysis revealed a tree with two distinct clades where one clade diverged into MoDiTPS2, 3, and 5 while the other clade consists of the remaining MoDiTPS1, 4 & 6 (Figure 1). The divergence of two clades is strongly supported by bootstrap value of 100 on the node of divergence. This observation is in agreement with Fischer et al. (2015) who suggested that fungal diterpene synthases essentially fall into two clades (Fischer et al., 2015). To assess if the observed phylogenetic expansion was distinct to diterpene synthases, a similar gene mining strategy for terpene synthase was employed for sesquiterpenes (C15) and sesterterpenes (C25). Here, the number of terpene synthases were consistent across all pathotypes (Supplementary Figures 3, 4). Thus, within the *Magnaporthe* terpenome of the currently sequenced strains, phylogenetic expansion seems to be limited to the DiTPSs.

### Comparison of Rice Blast DiTPSs to Characterized Fungal Diterpene Synthases in Ascomycete

**Figure 2:**
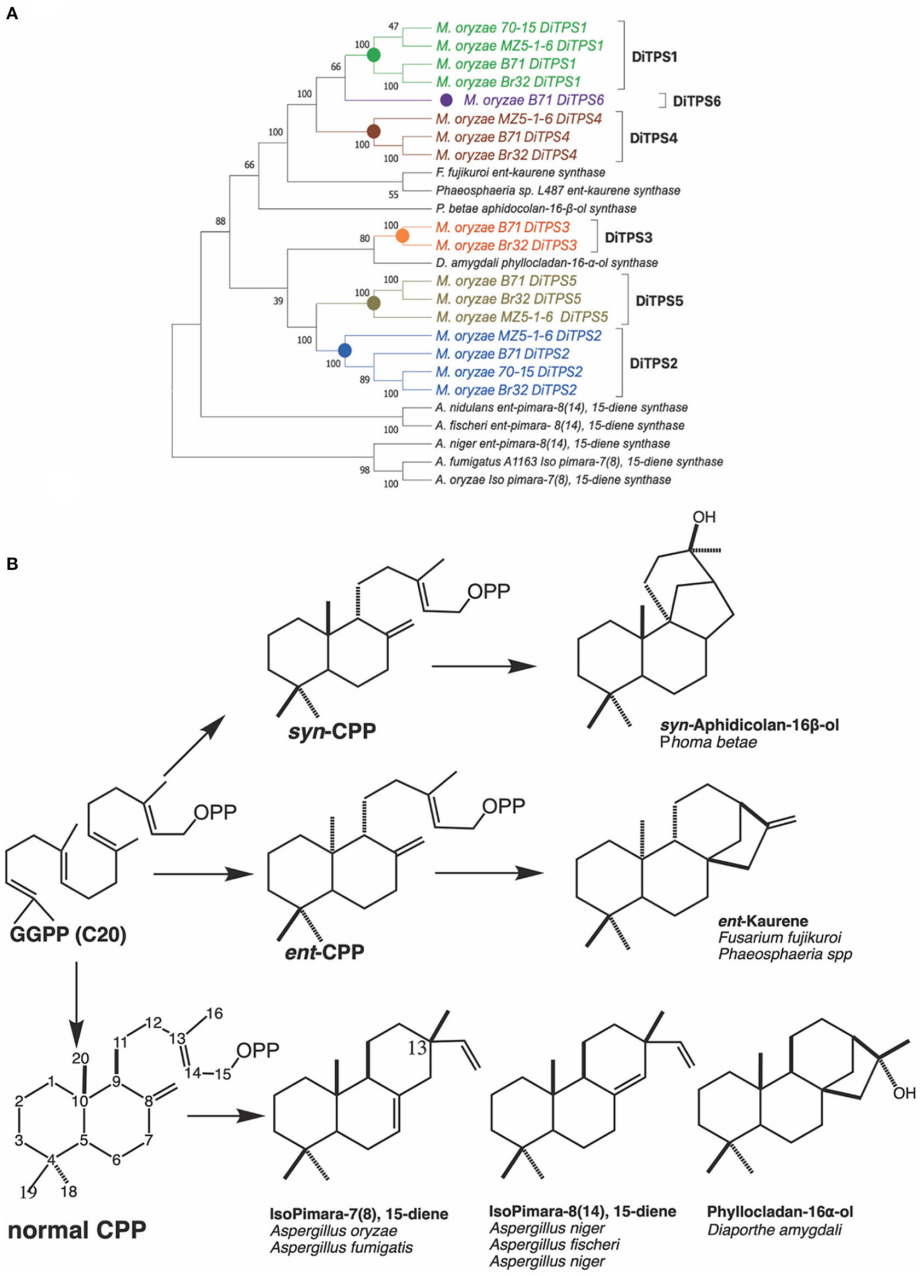
**(A)** Phylogenetic tree of biochemically characterized diterpene synthases in Ascomycete. Unrooted maximum likelihood tree showing relationship between *M. oryzae* diterpene synthases and characterized diterpene synthases from various species from Ascomycete. Characterized diterpene synthases are depicted in black and *M. oryzae* genes in various colors. Bootstrap values were determined with 100 replicates using MEGA X. Genbank accession numbers of the characterized diterpene synthases are available in Supplementary Table 4. **(B)** Structures of characterized diterpenes from Ascomycete. A summary of enzymatically characterized diterpene synthases from various fungi. Indicated is the intermediate stereoisomer of the Class II reaction and the resultant products mediated by the Class I active site. [*ent, syn*, and normal stereoisomers are dependent on the orientation of methyl and hydride substituents C9, C10 bond. Orientation of the methyl group at C13 differntiates between isopimaradiene (C13β) vs. pimaradiene (C13α)].

Fungal diterpene synthases have been traced back to an ancient horizontal gene transfer event from plants to Ascomycete (Fischer et al., 2015). We carried out phylogenetic analysis to compare how diterpene synthases from *M. oryzae* pathotypes relate to characterized diterpene synthases in Ascomycete (Figure 2). The diterpene synthases from genomes of three different pathotypes (70–15 reference genome, MZ5-1-6; fingermillet pathotype genome and B71; wheat pathotype genome) were aligned with previously characterized diterpene synthases. The analysis shows that *M. oryzae* diterpene synthases among these three distinct pathotypes are closely related to each other in each orthologous group. The phylogenetic tree strongly suggests (100 bootstrap value on the node of divergence) common ancestral origin of *ent*-kaurene synthases and MoDiTPS1, 4 and 6 clade. MoDiTPS2 and 5 have same node of divergence and do not group with any characterized DiTPS. However, DiTPS3 associates with a known phyllocladanol synthase (Toyomasu et al., 2008). Diterpene biosynthesis in *Magnaporthe* pathotypes provides a biological system to investigate the importance of chemical diversification in evolution and adaptation to its ecological niche.

### Biochemical Characterization of the Diterpene Synthases in *M. oryzae* Pathotypes

**Figure 3:**
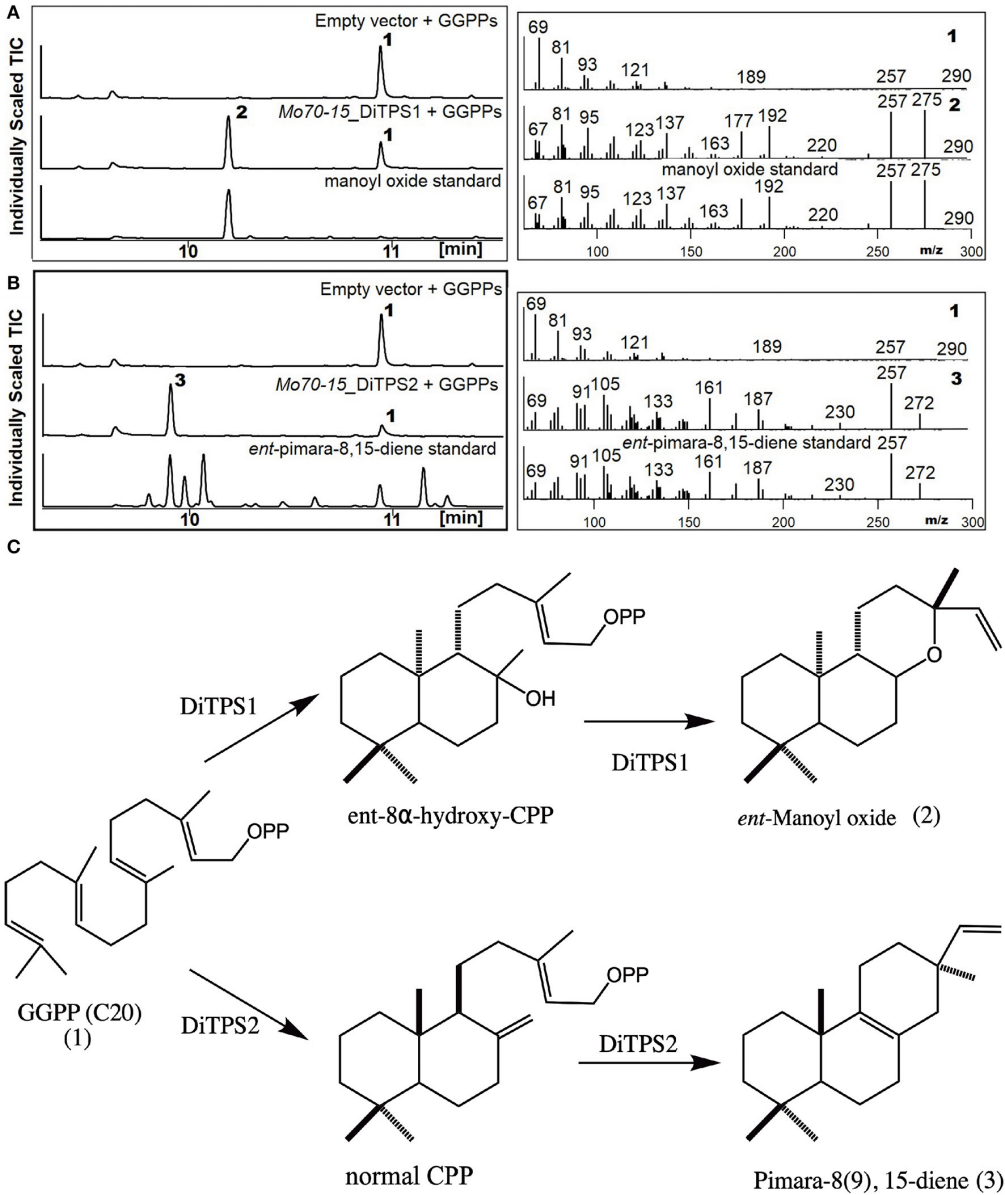
Biosynthesis of labdane diterpenes by *M. oryzae*. Analysis of resultant products by GC-MS of DiTPS co-expressed in *E. coli* optimized for the expression of diterpenes. The empty vector co-expressed with GGPP synthase was used as control and products verified by comparison to authentic standards. Chromatograms and mass spectra from GC-MS analysis of **(A)** DiTPS1 **(B)** DiTPS2 [*ent*-pimara8(9), 15-diene was used to confirm the metabolite followed by combinatorial expression with stereospecific enzymes to determine the intermediate stereochemistry] **(C)** Structures of the intermediate class II and class I products produced by *Magnaporthe*.

To determine the metabolites produced by the two core DiTPSs that are evolutionarily conserved in all pathotypes (i.e., MoDiTPS1 & 2), these were biochemically characterized using a previously described modular metabolic engineering system (Cyr et al., 2007; Morrone et al., 2010). Codon-optimized genes for each were expressed in *E. coli* engineered to make GGPP. The resultant diterpene scaffolds were confirmed by comparing both mass spectra and retention time to authentic standards (Figure 3). DiTPS1 carries out bifunctional cyclization resulting in manoyl oxide (Figure 3A). DiTPS2 resulted in formation of pimara-8(9),15-diene confirmed via comparison to an authentic standard (Figure 3B) (Karunanithi et al., 2020). The stereochemistry was determined as described below. The co-expression of these diterpene synthase genes with the precursor, GGPP in yeast strain ZXB also resulted in formation of same products (Supplementary Figure 5). Similarly, the expanded array of diterpene synthase from wheat (B71) and finger millet (MZ5-1-6) were expressed in the metabolic engineering system. Although B71 DiTPS1 was found to be nonfunctional in our expression system, interestingly DiTPS6 also produces manoyl oxide similar to DiTPS1, demonstrating functional conservation. DiTPS6 is paralogous to DiTPS1 with an 80% amino acid sequence identity suggesting parallel evolution (Supplementary Figure 6). In contrast, DiTPS3 which is in the same clade as DiTPS2, forms a yet to be identified product demonstrating duplication and neofunctionalization (Supplementary Figure 7). There was no activity observed for the remainder of the genes from wheat pathotype (B71) and fingermillet (MZ5-1-6) (Table 1, Supplementary Figure 6).

**Table 1:** Summary of characterization of diterpene synthases in *M. oryzae* pathotypes.

	**Diterpene scaffolds based on characterization**					
***M. oryzae* pathotypes characterized**	**MoDiTPS1**	**MoDiTPS2**	**MoDiTPS3**	**MoDiTPS4**	**MoDiTPS5**	**MoDiTPS6**
*Mo 70-15_Oryza sativa*			Absent	Absent	Absent	Absent
*B71_Triticum aestivum*	X		Active	X	X	
*MZ5-1-6_Eleusine coracana*	X		Absent	X	X	Absent

*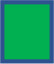 Manoyl oxide; 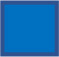 Pimara-8,15-diene; 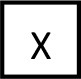 No product*.

### Determination of Class II Intermediates and Stereochemistry of MoDiTPS

To determine the diterpene scaffold intermediates generated by class II active site of the bifunctional diterpene synthases, the class I active site of the diterpene synthases were made inactive by mutation of first aspartate from the DDxxD motif to alanine (retains only the ability to make CPP in the class II active site). Subsequent co-expression of the class I mutants with GGPP resulted in production of the expected intermediate 8α-hydroxy-CPP by DiTPS1 and CPP for DiTPS2 (Supplementary Figure 8). To probe the substrate specificity of the class I active site and, hence, presumably also the stereochemistry of the corresponding class II product, the class II site was inactivated by mutation of the middle aspartate of the DxDD motif to alanine to eliminate CPS activity and fed various CPP (Figure 3) (Kawaide et al., 1997; Toyomasu et al., 2004, 2008; Bromann et al., 2012; Xu et al., 2017). The MoDiTPS1 class I active site did not react with normal 8α-hydroxy-CPP, nor any of the normal, e*nt* or *syn* stereoisomers of CPP (Supplementary Figure A). To assess whether the intermediate might be enantiomeric, we co-expressed the MoDiTPS1 class I mutant with stereospecific class I enzymes—i.e., an *ent*- specific kaurene synthase (AtKS) or normal specific abietadiene synthase (AgAS) (Mafu et al., 2015). Since only AtKS reacts with the MoDiTPS1 class II product *ent*-8α-hydroxy-CPP (Supplementary Figure A); MoDiTPS1 produces *ent*-manoyl oxide. The DiTPS2 class I active site reacts with normal CPP to produce pimara-8(9),15-diene, indicating that this is relevant stereoisomer of CPP produced by its class II active site. The DiTPS2 class I active site also reacts with *syn*-CPP resulting in unidentified products but does not form any products with *ent*-CPP (Supplementary Figure 8B). These results are consistent with previously characterized diterpene synthases from Ascomycete (Figure 2B).

### Biosynthetic Gene Cluster Analysis

From our analysis the putative MoDiTPS1 cluster possesses GGPP synthase (MGG_01971), oxidases/cytochromes P450 (MGG_01947, MGG_01950, MGG_01960), ligase (MGG_01951), and could possibly include a UDP glycosyl transferase (MGG_01961) (Supplementary Figure 9). The synteny results highlight that the BGCs are not conserved across the pathotypes as the cluster lacks a conserved transcription regulator (MGG_01946). The MoDiTPS1 cluster in genomes *B71* and *MZ5-1-6* may have variable regulator genes. The putative DiTPS2 cluster shows the presence of a complete and conserved biosynthetic gene cluster among *M. oryzae* pathotypes with precursor GGPP synthase (MGG_00026), oxidases and/or cytochromes P450 (MGG_00023, MGG_00024 & MGG_00031), dehydrogenase (MGG_00017); transcription factor (MGG_00032), and transporter (MGG_00040) (Supplementary Figure 9). The biochemical characterization also showed conservation of product formation [pimara-8(9), 15-diene] and functionality of MoDiTPS2 among *M. oryzae* pathotypes (Supplementary Figure 5). These results suggest a conserved role of MoDiTPS2 product in *M. oryzae* pathotypes. In addition, the MoDiTPS2 cluster is located next to another specialized metabolite cluster annotated as non-ribosomal peptide synthase, bassianolide synthase. It would be interesting to explore if a further functional correlation exists.

## Discussion

The two diterpene synthases (DiTPS1 and DiTPS2) are phylogenetically conserved across *Magnaporthe* pathotypes infecting rice and other grass species. From the literature, the two DiTPSs were transcriptionally upregulated under various conditions in rice and fingermillet (Saha et al., 2020, 2021). The presence of the normal pimara-8,15-diene producing DiTPS2 in all pathotypes indicates functional conservation. This conservation could imply an important role for these metabolites in in development and/or pathogenesis of the fungus, which still awaits experimental proof. Although DiTPS1 was only active in Mo70_15, the wheat pathotype B71 has acquired a paralog that also produces *ent*-manoyl oxide, suggesting a similar ability for the production of this diterpene as well.

Interestingly, the phylogenetic analysis across *Magnaporthe* pathotypes demonstrates an expansion of diterpene synthases that could be involved in adaptation of pathotypes for infection of other grasses. In particular, via intra-species duplication generated from the two core diterpene synthases (Figure 1). Terpenes are the largest group of specialized metabolites, and their diversity is a result of duplication and neofunctionalization events occurring with the genes involved in their biosynthesis (Cao et al., 2010; Peters, 2010). Our results provide examples of duplication resulting in parallel evolution, new function and non-active genes. The conservation of gene and function is comparable to previous reports in *Fusarium* species where terpene synthases were conserved across species, independent of host variety (Wiemann et al., 2013; Hoogendoorn et al., 2018). Notably, the BGC that encodes for the virulent factor gibberellic acid (GA) although present in several Fusarium strains is only functional in *F. fujikuroi*. Nevertheless, this research provides a strong basis for further studies to investigate if the observed phylogenetic expansion is limited to duplication, but generally results in pseudogenes rather than neofunctionalization or whether the observed inactivity is an artifact of the experimental process. Our results also highlight the need for production of both pimara-8,15-diene and *ent*-manoyl oxide activity, as well as hinting at a relationship to pathogenicity. Thus, our study defines the diterpene biosynthetic capability in *Magnaporthe* pathotypes and provides impetus to further investigate the importance of chemical diversification in evolution and adaptation of plant pathogenic fungi. As terpenes mediate diverse functional roles, the conserved production of at least these two core diterpenes suggests a biological role for these diterpenes in the rice blast fungus.

From the host plant perspective there is a strong diterpene-based chemical defense response in rice after infection by *M. oryzae* (Peters, 2006). We show in this study that the rice blast fungus also encodes functional diterpene synthases, but produces normal pimara-8,15-diene and *ent*-manoyl oxide, which are distinct from the diterpene scaffolds produced by rice (Figure 3C). These diterpene scaffolds are likely further decorated by downstream enzymes, including cytochromes P450 and reductases, to yield bioactive diterpenoids. The conservation of backbone formation and BGC suggest a conserved role of MoDiTPS2 in *M. oryzae* pathotypes. The BGC for MoDiTPS1 is more variable amongst the isolates which is consistent to the demonstrated variability in our biochemical analysis. The conservation of backbone formation and BGC suggest a conserved role of MoDiTPS2 in *M. oryzae* pathotypes. It would be worthwhile to confirm the BGC through experimental expression in heterologous hosts such as *Aspergillus* and yeast to not only identify the bioactive diterpenoids but understand the variability in the clusters.

As diterpenoids are essential for defense and resistance of rice to *M. oryzae*, the biochemical characterization described here provides information to enable experimental differentiation at the host-pathogen interface.

In conclusion, it is now evident that *M. oryzae* contains two diterpenes synthases that are evolutionarily conserved across different pathotypes, suggesting important roles for the resulting diterpenes. These labdane-related diterpenes produced by *Magnaporthe* are functionally distinct from those produced by rice. This research provides a strong basis for further studies of *in vivo* confirmation of gene function including testing whether the observed phylogenetic diversification confers an evolutionary advantage. Our study defines the diterpene biosynthetic capability in *Magnaporthe* pathotypes and provides insight to the importance of chemical diversification in evolution and adaptation.

## Data Availability Statement

The original contributions presented in the study are included in the article/Supplementary Material, further inquiries can be directed to the corresponding author.

## Author Contributions

AS performed all the experiments. AS and HY performed the computational analysis. AS and SM analyzed the data and wrote the article. SM designed the research and supervised the project. All authors commented on the results and manuscript. All authors contributed to the article and approved the submitted version.

## Funding

Financial support was provided by the Department of Biochemistry and Molecular Biology, UMass Amherst and Richard and Susan Smith Family Foundation, Newton MA to SM. AS was supported by Lotta M. Crabtree Fellowship.

## Conflict of Interest

The authors declare that the research was conducted in the absence of any commercial or financial relationships that could be construed as a potential conflict of interest.

## Publisher's Note

All claims expressed in this article are solely those of the authors and do not necessarily represent those of their affiliated organizations, or those of the publisher, the editors and the reviewers. Any product that may be evaluated in this article, or claim that may be made by its manufacturer, is not guaranteed or endorsed by the publisher.

## Abbreviations

DiTPS: diterpene synthase
GGPP: geranyl geranyl diphosphate
CPP: copalyl diphosphate.
