# Supplementary material for: Diterpene Biosynthesis in Rice Blast Fungus Magnaporthe
Source: Front Fungal Biol. 2022 Apr 12;3:869823. doi: 10.3389/ffunb.2022.869823 (PMC10512213; doi:10.3389/ffunb.2022.869823)
Supplement: Supplementary file 1 [file Data_Sheet_1.PDF]

Supporting information for

Diterpene biosynthesis in rice blast fungus *Magnaporthe*

Ayousha Shahi[1], Houlin Yu[1] and Sibongile Mafu [1,2]

[1]Plant Biology Graduate Program, University of Massachusetts-Amherst
[2]Department of Biochemistry and Molecular Biology, University of Massachusetts - Amherst
240 Thatcher Way, Life Science Laboratories, Amherst Massachusetts 01003

Corresponding author:
Mafu, Sibongile (smafu@umass.edu)

Content

Figure S1: Motifs and alignments of class I (DD/ExxD/E/H) and class II (DxDD) motifs of *M. oryzae* diterpene synthases with characterized diterpene synthases.

Figure S2: Flowchart for gene mining.

Figure S3: Molecular phylogenetic tree of sesquiterpene synthases of *M. oryzae* pathotypes.

Figure S4: Molecular phylogenetic tree of chimeric terpene synthases of *M. oryzae* pathotypes.

Figure S5: Functional characterization of *Mo_70-15* diterpene synthases in yeast.

Figure S6: Functional Characterization of MoDiTPS in *Magnaporthe* pathotypes infecting various grasses

Figure S7: Characterization of MoDiTPS3 from B71 wheat pathotype characterization of *Mo_B71_DiTPS3*.

Figure S8: Determination of class II intermediates and stereochemistry characterization of *Mo_70-15* diterpene synthases.

Figure S9: Synteny analysis of chromosomal regions containing MoDiTPS1 and MoDiTPS2 biosynthetic gene from three pathotype genomes, reference genome 70-15, finger millet pathotype, MZ5-1-6, and wheat pathotype B71

Table S1: Presence/Absence matrix of DiTPS in fourteen *M. oryzae* pathotypes used in Figure 1.

Table S2. List of primers

Table S3. List of gene constructs used as authentic standards

Table S4: Genbank accession numbers of characterized diterpene synthases in Ascomycete

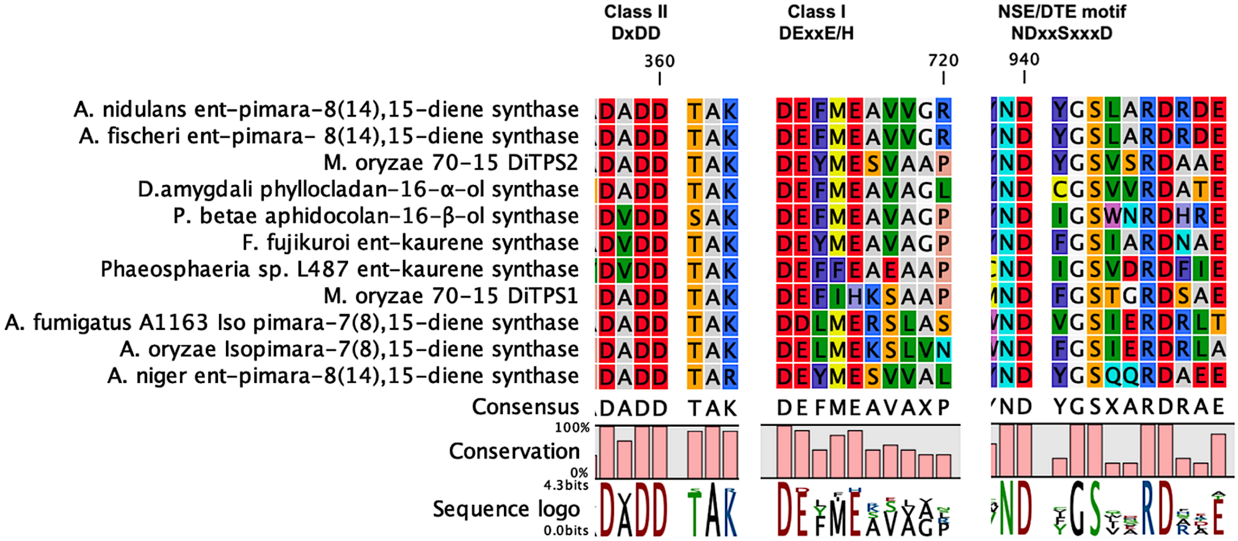

Figure S1. Motifs and alignments of class I (DD/ExxD/E/H) and class II (DxDD) motifs of *M. oryzae* diterpene synthases with characterized diterpene synthases.

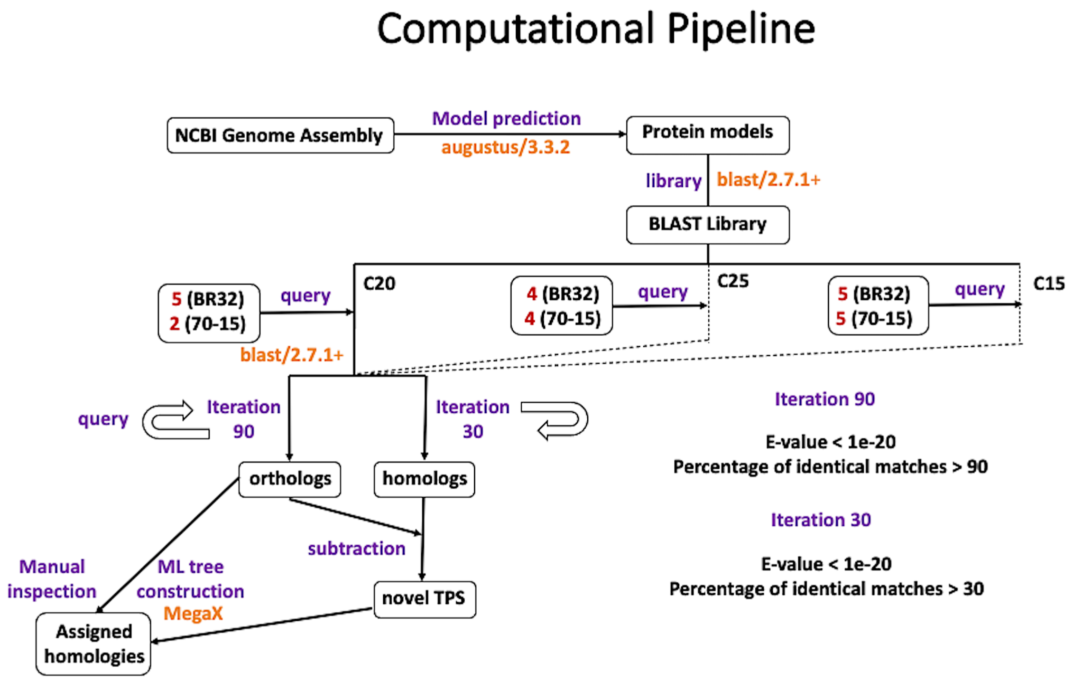

Figure S2. Flowchart for Gene Mining. Genome assemblies of 52 *Magnaporthe oryzae* strains[1] were downloaded from NCBI and their gene/protein models were predicted using Augustus/3.3.2 that was trained with *Magnaporthe grisea* gene model. A BLAST library of proteomes of all strains were made, and protein sequences of seven diterpene synthases (five from BR32 genome[2] and two from 70-15 genome from NCBI- MGG_01949 and MGG_14722) were queried against the library using blastp (blast/2.7.1+). The resulting matching sequences were filtered by retaining the matches with specific parameters (identity > 30% and e value < 1e-20). The filtered sequences were taken as the query again and searched against the BLAST library and the same filtering was applied. The process was iterated three times until no more matching sequences was found. The resulting sequences were manually screened for the presence of conserved motifs for respective terpene synthase and appropriate sequence lengths.

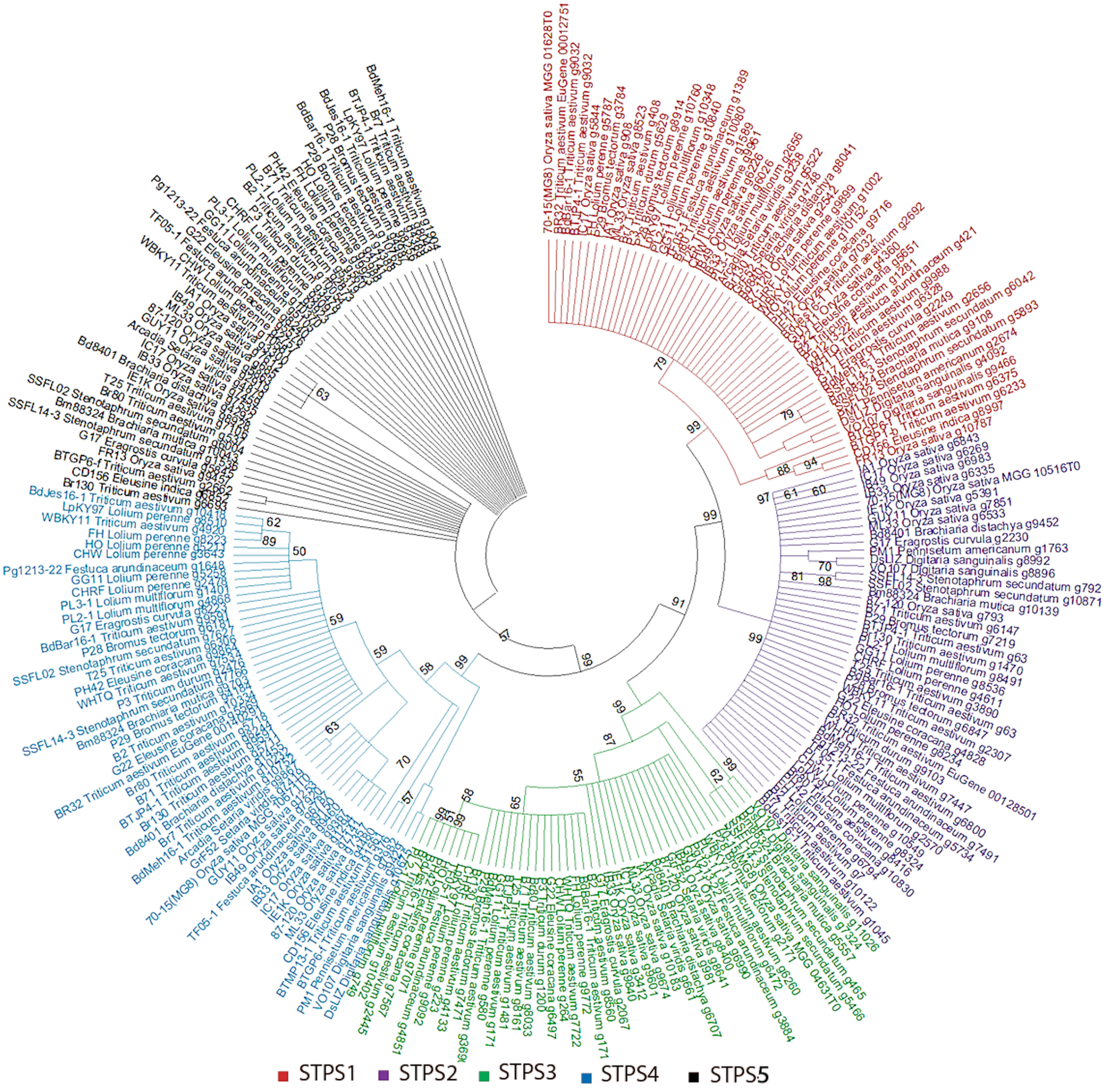

Figure S3. **Molecular phylogenetic tree of sesquiterpene synthases of *M. oryzae* pathotypes estimated using maximum likelihood (ML).** ML analysis was based on 234 enzymes from 52 genomes representing 12 pathotypes. The sequences with motifs- DDxxD, NSE/DTE and RxR and sequence lengths more than 220 amino acids were used for the analysis except for STPS3 that lacked RXR motifs. The sequence alignment was carried out using MUSCLE alignment tool and the ML tree was constructed using MEGAX software with default parameters. The numbers on branches indicate percent bootstrap support from 100 iterations and colored subtrees indicate distinct groups of sesquiterpene synthases as shown in the index.

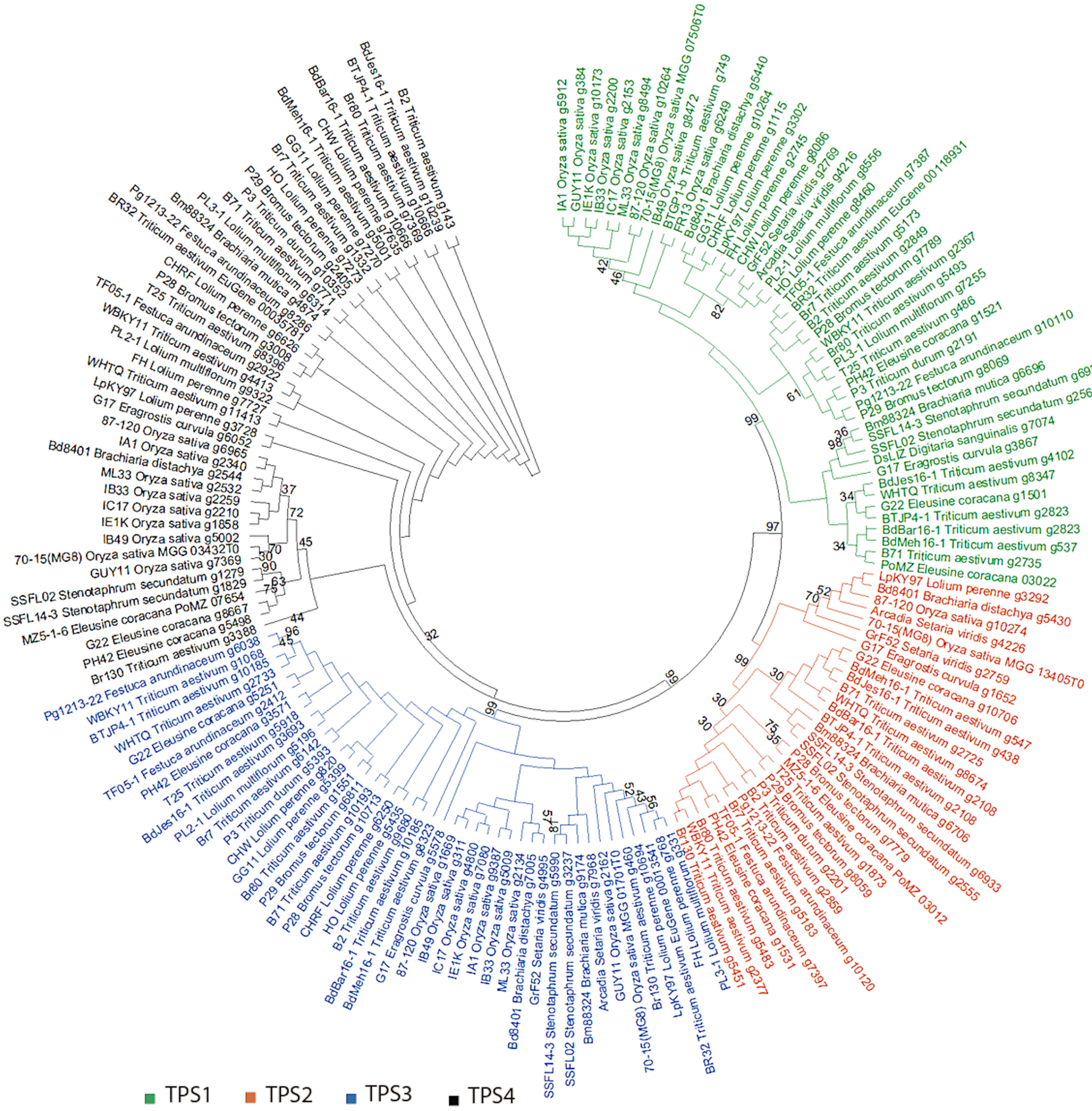

Figure S4. Molecular phylogenetic tree of chimeric terpene synthases of *M. oryzae* pathotypes estimated using maximum likelihood (ML). ML analysis was based on 164 enzymes from 52 genomes representing 12 pathotypes. The sequences with N-terminal motifs- D/ED/ExxD/E, NSE (N/HDxx(S/T)xxxD/E) and C-terminal motifs- DDxxD and DDxxN were used for the study. The TPS1 possessed NSY/A motifs instead of NSE/DTE motif. The sequences less than 600 aa were excluded. The sequence alignment was carried out using MUSCLE alignment tool and the ML tree was constructed using MEGAX software with default parameters. The numbers on branches indicate percent bootstrap support from 100 iterations and colored subtrees indicate distinct groups of chimeric terpene synthases as shown in the index.

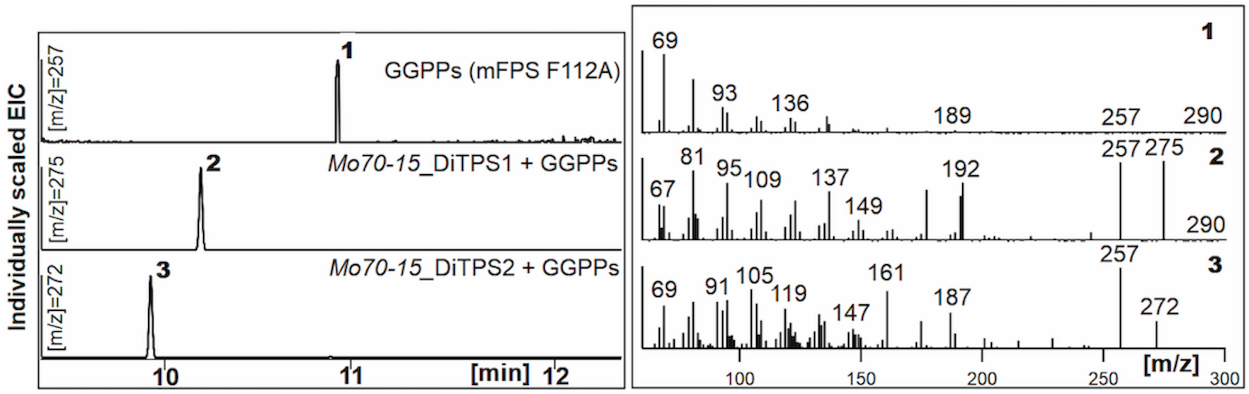

Figure S5. Functional characterization of *Mo_70-15* diterpene synthases in yeast. Extracted ion chromatograms (EIC) and mass spectra of extract from yeast (strain) co-transformed with Geranylgeraniol (GGPP) synthase (mFPS F112A) and DiTPS1/DiTPS2. Geranylgeraniol **1,** manoyl oxide **2**, pimara-8,15-diene **3**.

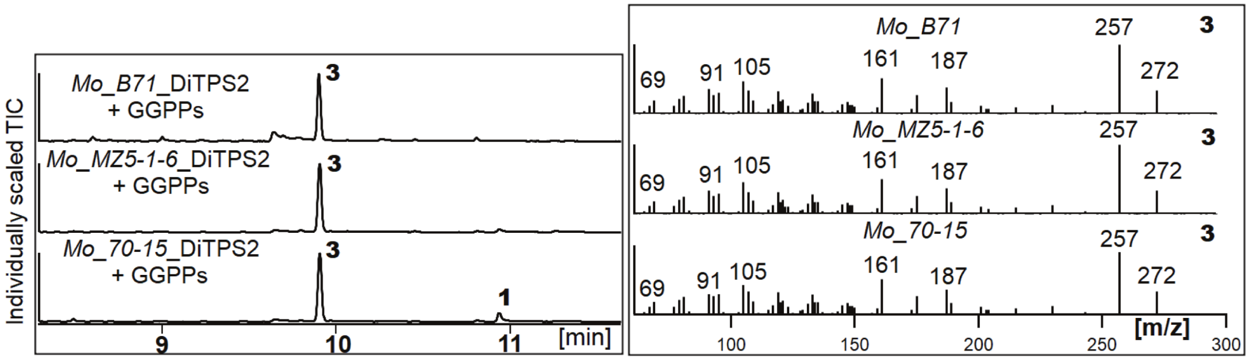

Figure S6: I. Functional Characterization of MoDiTPS in *Magnaporthe* pathotypes infecting various grasses. Total ion chromatograms (TIC) and mass spectra of extract from co-expression of **DiTPS2 from *B71, MZ5-1-6 and 70-15* pathotypes** with the substrate GGPP synthase Geranylgeraniol **1**, pimara-8,15-diene

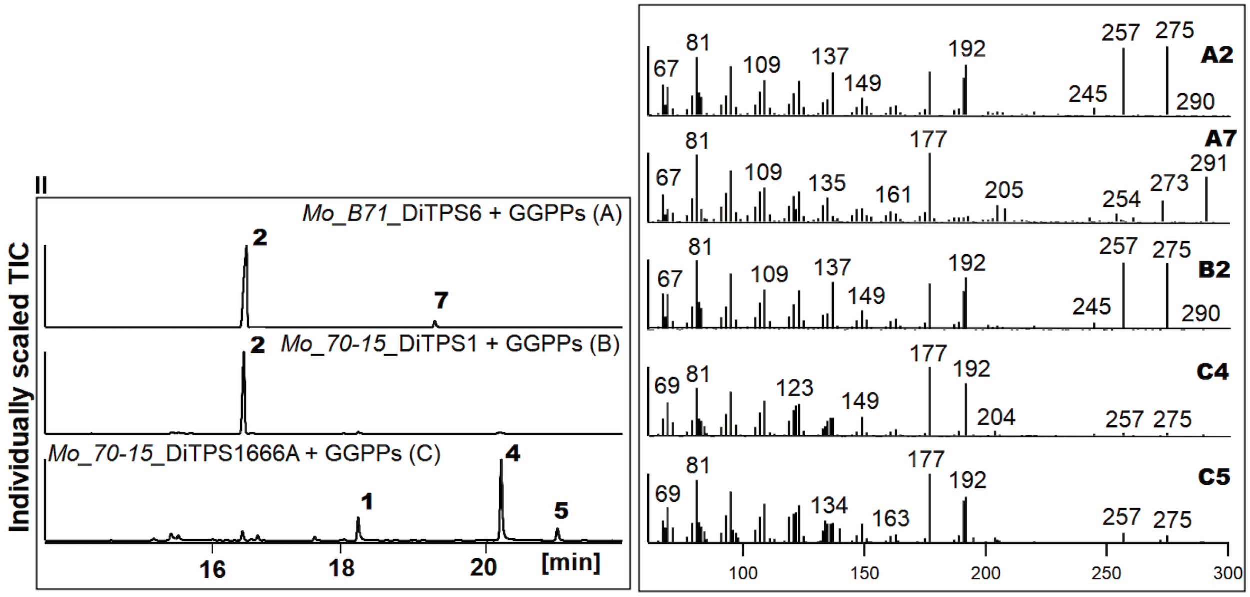

Figure S6: II. Functional characterization of *MoB71_Triticum_aestivum*_DiTPS6. Total ion chromatograms (TIC) and mass spectra of extract from co-expression of ***MoB71_Triticum_aestivum*_DiTPS6** with the substrate GGPP synthase compared to *Mo_70-15*_DiTPS1 and its class I mutant intermediate. Geranylgeraniol **1**, manoyl oxide **2**, 8-hydroxy-CPP **4**, **5**, unknown product **7**.

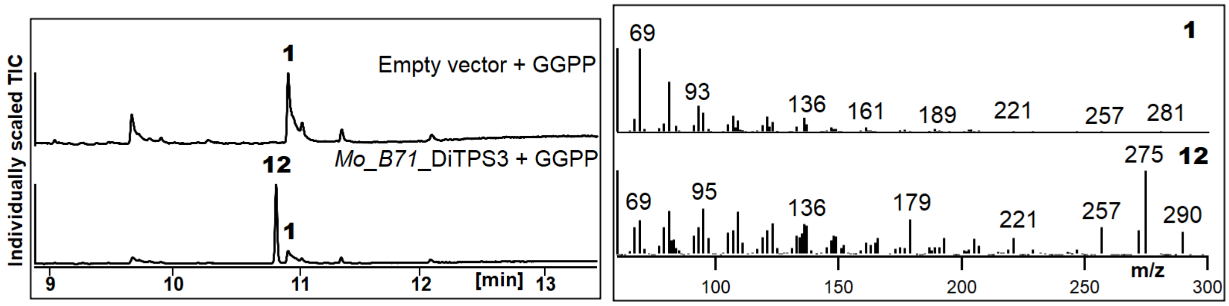

Figure S7. Characterization of MoDiTPS3 from B71 wheat pathotype. I. Total ion chromatograms (TIC) and mass spectra of extract from *E. coli* (C41pIRS*)* co-expressing GGPP synthase and *MoB71*_DiTPS3. Geranylgeraniol **1**, unknown product **12**.

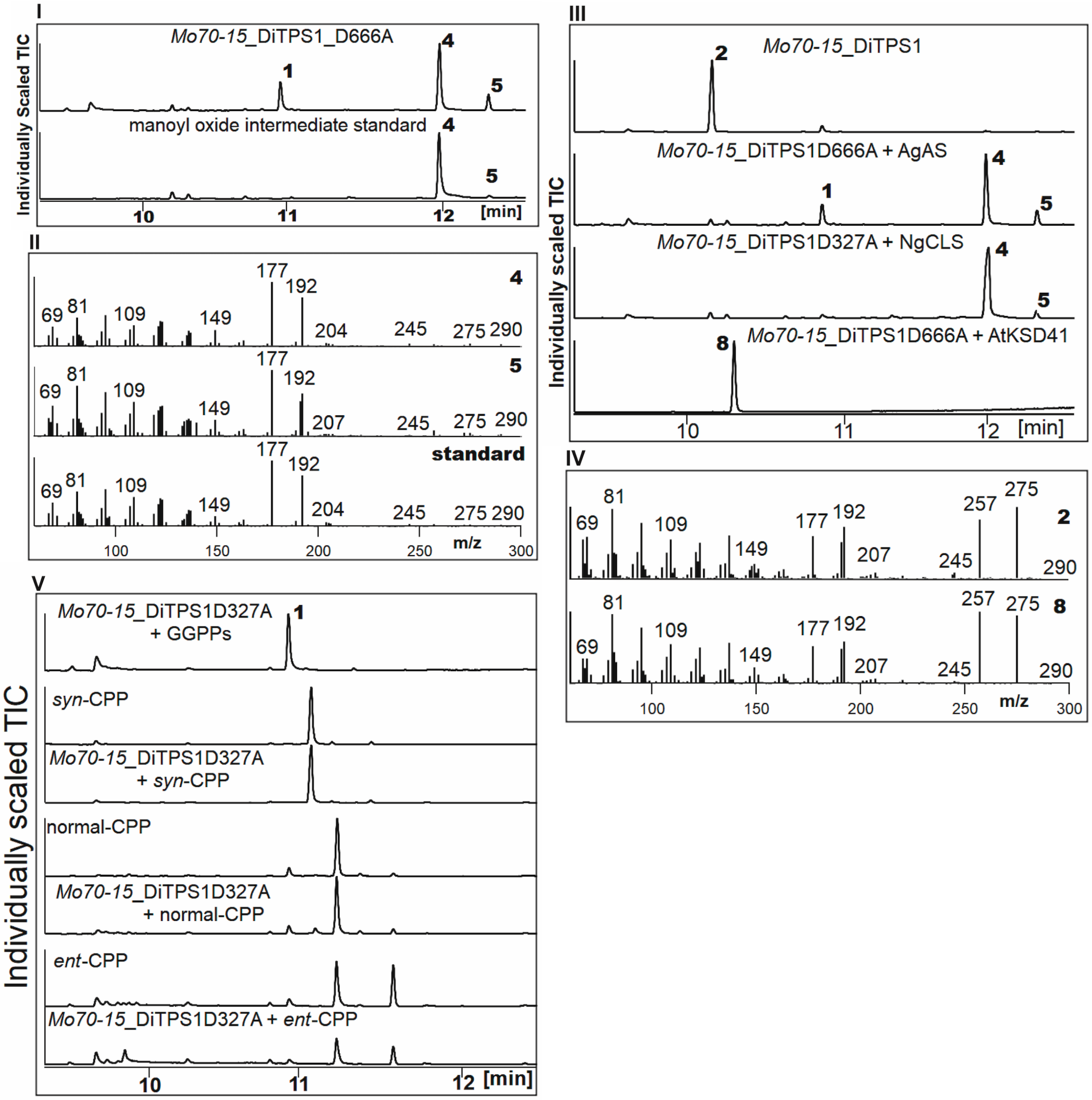

Figure S8a. Characterization of class II intermediate of MoDiTPS1. I. Total ion chromatograms (TIC) of extract from *E. coli* (C41pIRS*)* co-expressing GGPP synthase and *Mo70-15*_DiTPS1D666A (class I mutant). Geranylgeraniol **1**, 8-hydroxy-CPP **4**, **5.** The intermediates' peaks and mass spectra were compared with the standard NgCLS. **II**. Mass spectra of the products 4, 5 and standard. **III.** Total ion chromatograms (TIC) of *E. coli* extract from co-expression of *Mo70-15*_DiTPS1 class I (D666A) and class II (D327A) mutants with their complementary enzyme from the enzyme pair used as standard – AgASD404A and NgCLS and ent- hydroxy CPP standard- AtKSD41. Manoyl oxide **2**, Unknown product **8***. *Note: Although DiTPS1D666A reacts with en- specific AtKS it results in compound 8 (13-epi manoyl oxide) which has a different retention time.* **IV**. Mass of the products 2 and 8. **V**. Total ion chromatograms (TIC) of extract from co-expression of DITPS1 class II (D327A) mutants with stereoisomers of copalyl diphosphate synthases (*syn*, normal and *ent* CPS) in *E. coli* (C41pIRS).

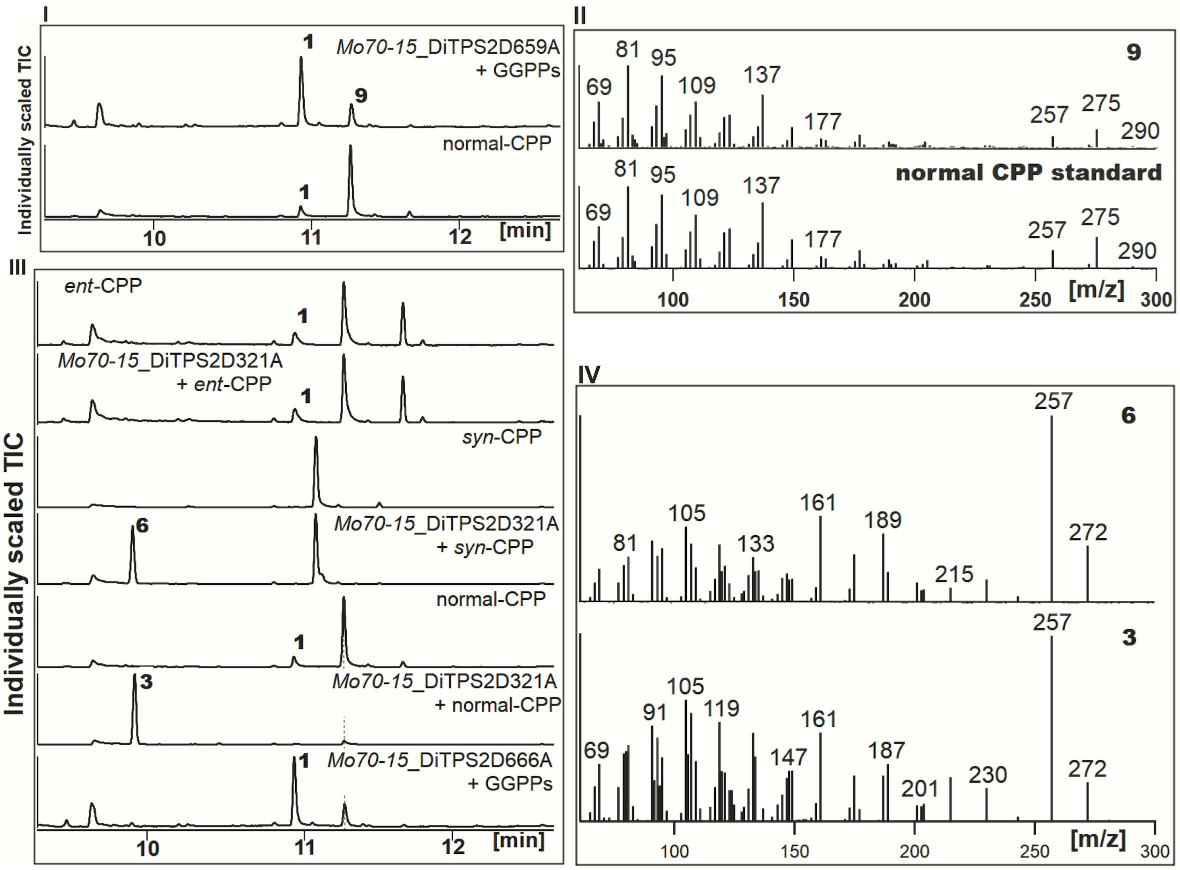

Figure S8b. Characterization of class II intermediate of MoDiTPS2. I. Total ion chromatograms (TIC) of extract from *E. coli* (C41pIRS*)* co-expressing GGPP synthase and *Mo70-15*_DiTPS2D659A (class I mutant). Geranylgeraniol **1**, normal-CPP **9.** The intermediates' peaks and mass spectra were compared with the standard pGGnC. **II**. Mass spectra of the product 7 and standard. **III.** Total ion chromatograms (TIC) of *E. coli* extract from co-expression of *Mo70-15*_DiTPS2 class II (D321A) mutants with stereoisomers of copalyl diphosphate synthases (*syn*, normal and *ent* CPS) in *E. coli* (C41pIRS) to identify MoDiTPS2 intermediate and its stereochemistry. Geranylgeraniol **1**, pimara-8,15-diene **3**, unknown product **6**. **IV**. Mass spectra of the unknown product **6**.

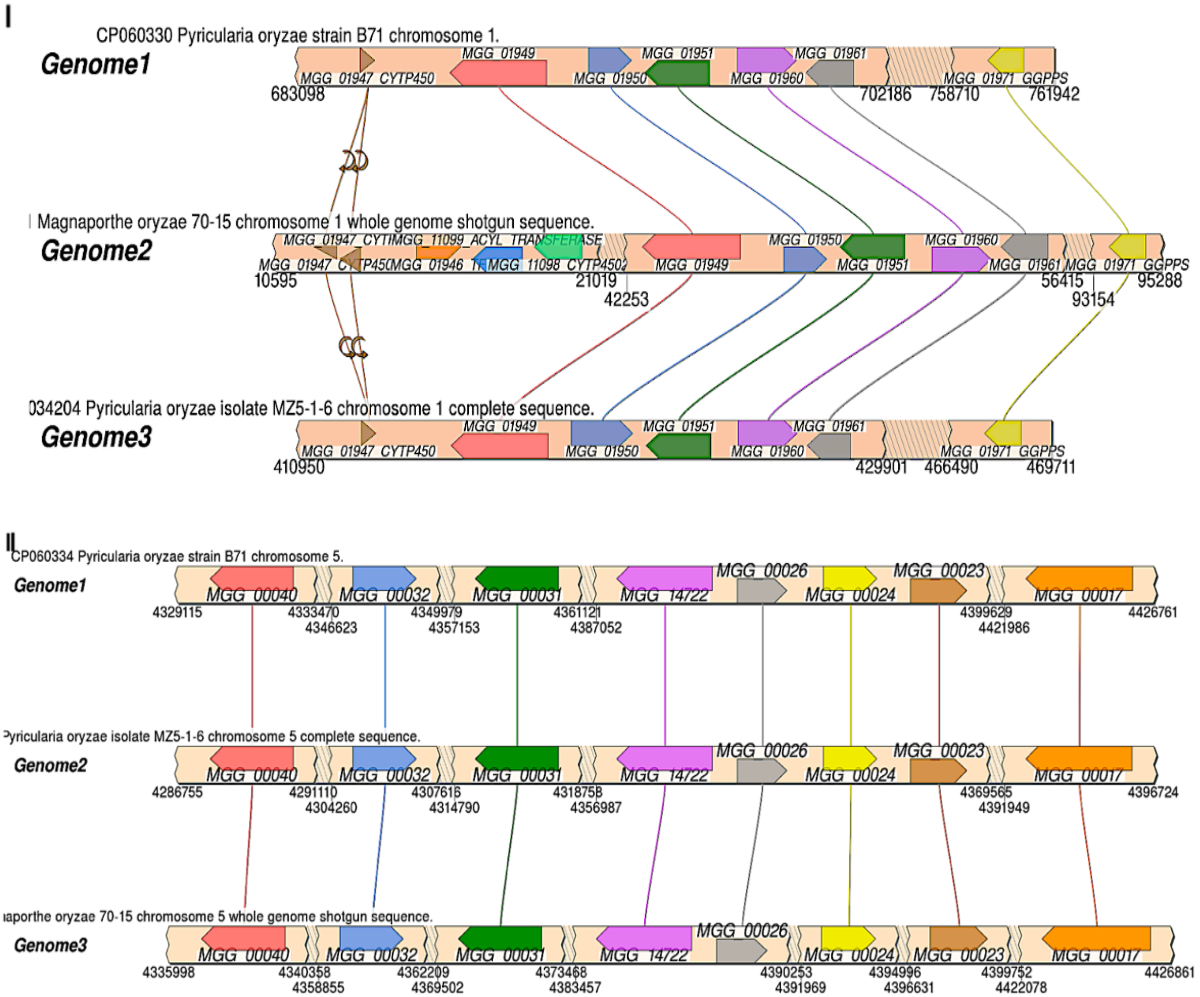

Gene Description	DiTPS1	DiTPS2
GGPP	MGG_01971	MGG_00026
Diterpene Cyclase	MGG_01949	MGG_14722
Cytochrome P450/ Oxidoreductase/ hydroxylase	MGG_01947 MGG_01950 MGG_01960	MGG_00023 MGG_00024 MGG_00031
Dehydrogenase		MGG_00017
Ligase	MGG_01951	
Transporter		MGG_00040
Transcription Factor	MGG_01946	MGG_00032

Figure S9. Synteny analysis of chromosomal regions containing MoDiTPS1 and MoDiTPS2 biosynthetic gene from three pathotype genomes, reference genome 70-15, finger millet pathotype, MZ5-1-6, and wheat pathotype B71. I. Synteny analysis of biosynthetic genes of MoDiTPS1 (MGG_01949), II. Synteny analysis of biosynthetic genes of MoDiTPS2 (MGG_14722) III. Putative biosynthetic gene clusters of MoDITPS1 and MoDiTPS2.

Table S1. Presence/Absence matrix of DiTPS in fourteen *M. oryzae* pathotypes used in Figure 1.

		**Diterpene synthases**					
**Hosts**	***M. oryzae* genomes**	DiTPS1	DiTPS2	DiTPS3	DiTPS4	DiTPS5	DiTPS6
Oryza sativa	*Mo 70-15*			X	X	X	X
	Guy11			X	X	X	X
Triticum aestivum	*Br32*						X
	B71						
Setaria viridis	*GrF52*	X					X
Setaria italica	*US71*	X			X	X	X
Eleusine coracana	*MZ5-1-6*			X			X
	G22						X
Brachiaria mutica	*Bm88324*						X
Brachiaria distachya	*Bd8401*	X					X
Bromus tectorum	*P28*		X				X
	P29						
Lolium perenne	*GG11*						X
	LpKY97						X

Present X Absent

Table S2. List of primers

Primers	Vector	Primer Sequence
MO_01949 F	pET28b	AATGGGTCGCGGATCATGAAACGTGCAAAAAGCGTTCC
MO_01949 R	pET28b	GTGCGGCCGCAAGCTATCCAGTTCGGCTGCACC
MO_14722 F	pET28b	AATGGGTCGCGGATCATGGGTAGCCTGACCTTTCC
MO_14722 R	pET28b	GTGCGGCCGCAAGCTCATATTTGCTGCTGCGGC
MO_01949 F	pESC-URA	CACTAAAGGGCGGCCATGAAACGTGCAAAAAGCGTTCC
MO_01949 R	pESC-URA	ATCCATCGATACTAGATCCAGTTCGGCTGCACC
MO_14722 F	pESC-URA	CACTAAAGGGCGGCCATGGGTAGCCTGACCTTTCC
MO_14722 R	pESC-URA	ATCCATCGATACTAGCATATTTGCTGCTGCGGC
1949 D666A F	pET28b	CGCTTTTATGAATAAATTCA**G**CGGCCTGATAGCTCAGCAT
1949 D666A R	pET28b	ATGCTGAGCTATCAGGCCG**C**TGAATTTATTCATAAAAGCG
1949 D327A F	pET28b	TGCACCGGATGCCG**C**TGATACGGCCAAAG
1949 D327A R	pET28b	CTTTGGCCGTATCA**G**CGGCATCCGGTGCA
14722 D659A F	pET28b	CGCTTTCCATATATTCA**G**CACCCTGATAGCTCAGC
14722 D659A R	pET28b	GCTGAGCTATCAGGGTG**C**TGAATATATGGAAAGCG
14722 D321A R	pET28b	GCATTATGGCAGATGCAG**C**TGATACAGCAAAAACCCT
14722 D321A F	pET28b	AGGGTTTTTGCTGTATCA**G**CTGCATCTGCCATAATGC

Table S3. List of gene constructs used as authentic standards

Enzyme	Gene constructs used as standards	Product
DiTPS1	AgASD404A + NgCLS[3]	Manoyl oxide
	AtKSD41	*ent*-8-hydroxy CPP
DiTPS2	SaDTS + normal CPP + GGPP [4]<br><br>SiTPS8+ZmAn2[5]	Isopimara- 8,15-diene,<br><br>*ent* pimara-8,15-diene
	ZmAn2	*ent*-CPP
	pGGsC	*syn*-CPP[6]
	pGGnC	normal-CPP

Table S4. Genbank accession numbers of characterized diterpene synthases.

Characterized diterpene synthases	Genbank accession numbers	References
M. oryzae 70-15 manoyl oxide synthase	XP_003708691	This study
M. oryzae 70-15 pimara-8,15-diene synthase	XP_003719120	This study
A. fumigatus A1163 Isopimara-7,15-diene	EDPS2216	Xu et al., 2017
F. fujikuroi ent-kaurene synthase	CPSKS_GIBFU	Toyomasu et al, 2000
Phaesophaeria sp. L487 ent-kaurene synthase	CPSKS_PHASA	Kawaide et al., 1997
P. betae aphidicolan-16b-ol synthase	BAD29971	Toyomasu et al. 2004
D. amygdali phyllocladan-16a-ol synthase	BAG30961	Toyomasu et al., 2008
A. nidulans FGSCA4 ent-pimara-8(14),15-diene synthase	XP_659198	Bromann et al., 2012
A. niger CBS513.88 ent-pimara-8(14),15-diene synthase	XP_001398730	Xu et al., 2017
A. oryzae Isopimara-7,15-diene synthase	XP_001820661	Xu et al., 2017
A. fischeri NRRL 181 ent-pimara-8(14),15-diene synthase	XP_001264196	Xu et al., 2017

References

(1) Gladieux, P.; Condon, B.; Ravel, S.; Soanes, D.; Maciel, J. L. N.; Nhani, A.; Chen, L.; Terauchi, R.; Lebrun, M.-H.; Tharreau, D.; Mitchell, T.; Pedley, K. F.; Valent, B.; Talbot, N. J.; Farman, M.; Fournier, E. Gene Flow between Divergent Cereal- and Grass-Specific Lineages of the Rice Blast Fungus <Em>Magnaporthe Oryzae</Em> *MBio* **2018**, *9* (1), e01219-17. https://doi.org/10.1128/mBio.01219-17.

(2) Chiapello, H.; Mallet, L.; Guérin, C.; Aguileta, G.; Amselem, J.; Kroj, T.; Ortega-Abboud, E.; Lebrun, M. H.; Henrissat, B.; Gendrault, A.; Rodolphe, F.; Tharreau, D.; Fournier, E. Deciphering Genome Content and Evolutionary Relationships of Isolates from the Fungus Magnaporthe Oryzae Attacking Different Host Plants. *Genome Biol. Evol.* **2015**, *7* (10), 2896–2912. https://doi.org/10.1093/gbe/evv187.

(3) Mafu, S.; Potter, K. C.; Hillwig, M. L.; Schulte, S.; Criswell, J.; Peters, R. J. Efficient Heterocyclisation by (Di)Terpene Synthases. *Chem. Commun.* **2015**, *51* (70), 13485–13487. https://doi.org/10.1039/c5cc05754j.

(4) Xu, M.; Hillwig, M. L.; Lane, A. L.; Tiernan, M. S.; Moore, B. S.; Peters, R. J. Characterization of an Orphan Diterpenoid Biosynthetic Operon from Salinispora Arenicola. *J. Nat. Prod.* **2014**, *77* (9), 2144–2147. https://doi.org/10.1021/np500422d.

(5) Karunanithi, P. S.; Berrios, D. I.; Wang, S.; Davis, J.; Shen, T.; Fiehn, O.; Maloof, J. N.; Zerbe, P. The Foxtail Millet (Setaria Italica) Terpene Synthase Gene Family. *Plant J.* **2020**, *103* (2), 781–800. https://doi.org/10.1111/tpj.14771.

(6) Cyr, Anthony, Wilderman Ross, Determan, M. and P. R. J. A Modular Approach for Facile Biosynthesis of Labdane-Related Diterpenes. *J. Am. Chem. Soc.* **2007**, *129*, 6684–6685.
